# Clinical characteristics and preliminary discriminatory value of low triiodothyronine syndrome in patients with peritoneal dialysis-associated peritonitis: A cross-sectional study

**DOI:** 10.1097/MD.0000000000051183

**Published:** 2026-10-23

**Authors:** Jiaxin Liu, Liqiang Lv, Junyuan Xiao, Rui Wu, Chuxin Chen, Yanqiang Peng

**Affiliations:** a Department of Nephrology, The First Affiliated Hospital of Shantou University Medical College, Shantou, Guangdong Province, China.

**Keywords:** Inflammatory markers, Low T3 syndrome, Neutrophil-to-lymphocyte ratio, Peritoneal dialysis-associated peritonitis, Refractory peritonitis

## Abstract

This study aimed to examine the clinical characteristics and preliminarily evaluate the discriminatory value of low triiodothyronine (T3) syndrome in patients with peritoneal dialysis-associated peritonitis (PDAP). Patients admitted for PDAP between January 2020 and May 2022 were categorized into a tested group and an untested group based on whether thyroid function testing was performed at peritonitis onset. The tested group was further subdivided into a low T3 syndrome subgroup and a normal thyroid function subgroup for comparative analysis of clinical characteristics. Correlations between free triiodothyronine (FT3) and other indicators were analyzed. The preliminary diagnostic discriminative value of PDAP complicated by low T3 syndrome was then assessed. Among 181 PDAP patients, 11 were excluded for not meeting the criteria, resulting in 69 patients in the tested group and 101 in the untested group. Baseline characteristics were comparable between the 2 groups, except for the proportion of refractory peritonitis. In the tested group, 26 were in the low T3 syndrome subgroup and 43 in the normal thyroid function subgroup. Compared to the normal group, the low T3 syndrome group had significantly higher levels of WBC, neutrophil count, PCT, and CRP, as well as a higher proportion of refractory peritonitis (all *P < *.05). FT3 at peritonitis onset was positively correlated with baseline FT3 but negatively correlated with WBC, neutrophil count, PCT, CRP, and NLR (all *P < *.05). Multivariate analysis showed baseline FT3 and WBC remained as factors independently associated with FT3 levels at peritonitis onset (*B* = 0.410, 95% CI 0.214–0.605 and *B *= −0.055, 95% CI −0.084 to −0.026, both *P* < .001). Compared to combinations involving PCT or CRP, baseline FT3 combined with WBC, neutrophil count, or NLR demonstrated numerically higher AUC values for PDAP patients with low T3 syndrome, specifically 0.750, 0.744, and 0.721 (*P* < .05). PDAP patients complicated by low T3 syndrome exhibited elevated levels of inflammatory markers and a higher proportion of refractory peritonitis. Baseline FT3 and WBC were independently associated with FT3 levels at peritonitis. The combination of baseline FT3 and inflammatory markers showed preliminary discriminatory value for identifying low T3 syndrome in patients with PDAP.

## 1. Introduction

In recent years, the number of patients undergoing peritoneal dialysis (PD) has been steadily rising worldwide. Approximately 11% of end-stage renal disease (ESRD) patients opt for PD as a renal replacement therapy, particularly in China, Mexico, the United States, and Thailand.^[1]^ According to data from the Chinese National Renal Data System (CNRDS), the number of patients receiving PD in China increased by 2.49-fold between 2012 and 2021. Peritoneal dialysis-associated peritonitis (PDAP) is a common complication that can result in increased hospitalization rates, dialysis failure, and even death. A global epidemiological survey indicated that the incidence of PDAP was highest in Asia-Pacific countries, followed by Europe, the Middle East, and Africa, with the lowest incidence in the United States.^[2]^ A low triiodothyronine (T3) level is associated with inflammation, malnutrition, and comorbidity burden in patients with chronic diseases. It is also a risk factor for cardiovascular events and mortality.^[3]^ Several studies have reported an association between low T3 levels and inflammation in dialysis patients.^[4,5]^ This study aimed to investigate the clinical characteristics of low T3 syndrome in PDAP patients and to evaluate the preliminary discriminatory value of clinical indicators for this condition.

## 2. Methods

### 2.1. Patients

This study included patients with PDAP admitted to our hospital between January 2020 and May 2022. PDAP was diagnosed based on at least two of the following criteria: abdominal pain and/or cloudy peritoneal dialysis fluid; white blood cell count in peritoneal effluent > 0.1 × 10^9^/L; positive microbiological culture of peritoneal effluent.^[6]^ Low T3 syndrome is defined as low T3 levels with normal thyroid-stimulating hormone (TSH) and thyroxine (T4).^[7]^ The exclusion criteria were as follows: age under 18 or over 80 years; PD treatment duration of <3 months; presence of thyroid diseases, malignant tumors, or thyroidectomy; missing laboratory data; history of medication use affecting thyroid function, such as lithium carbonate, iodide, or amiodarone; and complications during hospitalization, including acute myocardial infarction, shock, or active hemorrhage.

To evaluate potential selection bias for the 2 groups with respect to baseline characteristics and treatment outcomes, patients were stratified into a tested group and an untested group according to whether thyroid function tests were performed at the onset of peritonitis. Based on free triiodothyronine (FT3) levels, the tested group was then further subdivided into the normal thyroid function group and the low T3 syndrome group.

Each patient with PDAP received standard antibiotic regimens in accordance with the International Society for Peritoneal Dialysis guidelines.^[6]^ Empirical antibiotic therapy with intraperitoneal cefazolin and ceftazidime was initiated to provide coverage against both Gram-positive and Gram-negative organisms, with subsequent adjustments based on culture results and clinical response. In cases of inadequate clinical response or bacterial resistance, therapy was switched to vancomycin or carbapenems.Tuberculous peritonitis was treated with 4 antituberculosis drugs.

Refractory peritonitis is defined as cloudy peritoneal effluent or a white blood cell count > 0.1 × 10^9^/L persisting after 5 days of appropriate antibiotic treatment. The outcomes for peritonitis were categorized as peritonitis cure, defined as the resolution of clinical signs and symptoms with clear peritoneal effluent after a complete course of antibiotics, and treatment failure, defined as catheter removal or death. The catheter was removed from patients for whom the antibiotic treatment remained ineffective or who were diagnosed with fungal peritonitis.

### 2.2. Clinical data collection and laboratory measurement

Clinical data, including age, sex, primary disease, and peritonitis pathogens, were collected from the participants. The duration of PD, peritonitis outcomes (cure or failure), proportion of refractory peritonitis, and body mass index (BMI) were recorded. Laboratory data included serum albumin (ALB), C-reactive protein (CRP), procalcitonin (PCT), peripheral white blood cell count (WBC), hemoglobin, neutrophil count, peritoneal effluent white blood cell count, neutrophil-to-lymphocyte ratio (NLR), and platelet-to-lymphocyte ratio (PLR).

Given that most patients with PDAP had hypoalbuminemia, which may potentially result in decreased levels of total T3 and total T4, this study recorded only FT3 and FT4. In this study, thyroid function was assessed using chemiluminescence assays. The normal reference intervals for FT3, FT4, and TSH are 3.24–6.47 pmol/L, 7.64–16.03 pmol/L, and 0.38–5.33 μIU/ml, respectively. Low T3 syndrome was defined as FT3 below 3.24 pmol/L alongside normal FT4 and TSH levels. FT3, FT4, and TSH were measured at PDAP onset. These indicators were also measured at the time point closest to PDAP onset and were used as baseline values. The interval from the baseline FT3 measurement to PDAP onset was also recorded.

### 2.3. Statistical analysis

According to the category of different data, baseline demographics and laboratory data were recorded as mean ± standard deviation, median (25th−75th percentile), or frequency (percentage). The normality of the distribution of parameters was tested using the Shapiro–Wilk test. Comparisons between 2 groups were performed by Student’s t tests, Mann-Whitney U tests, or chi-square (χ^2^) tests as appropriate. Associations between serum FT3 levels and normally distributed continuous variables were analyzed using Pearson’s correlation analysis, and those involving asymmetrically distributed variables were analyzed using Spearman’s correlation analysis. Variables with *P* < .10 in univariate linear regression analysis for inclusion in multivariate linear regression models using a stepwise method. The overall significance of the final multivariate linear regression model was assessed using analysis of variance; homoscedasticity was assessed using a plot of standardized residuals versus standardized predicted values; normality was assessed using the P-P plot; and multicollinearity among the independent variables was assessed using variance inflation factor (VIF) analysis. Multivariate linear regression analysis with 95% confidence intervals was used to identify factors independently associated with FT3 levels at PDAP. Receiver operating characteristic (ROC) curve analysis was performed to evaluate the preliminary discriminatory value for PDAP complicated by low T3 syndrome. A binary logistic regression model was used to develop combined models by combining baseline FT3 levels and inflammatory markers to improve diagnostic accuracy. The preliminary discriminatory probabilities derived from the above regression models were subsequently utilized as novel composite variables to generate ROC curves for the combined diagnostic assessment. A *P*-value < .05 was considered statistically significant.

## 3. Results

A total of 181 patients with PDAP were identified. After excluding 11 who met the exclusion criteria (including 3 patients with PD duration <3 months, 2 patients with thyroidectomy, 1 patient with hyperthyroidism, 1 patient with hypothyroidism, 2 patients with shock, 1 patient with a malignant tumor, and 1 patient with myocardial infarction), 170 patients were enrolled in the study. Of these, 101 patients did not undergo thyroid function assessment at peritonitis onset (termed the untested group), while the remaining 69 did (termed the tested group) (Fig. 1). As thyroid function testing was not a routine examination, it was performed only at the discretion of individual physicians. To assess potential selection bias, baseline characteristics including sex, age, PD duration, ALB, peritoneal effluent white blood cell count, peripheral WBC, PCT, CRP, the proportion of refractory peritonitis cases, and peritonitis outcomes were compared between the 2 groups. The analysis revealed no statistically significant differences in most baseline characteristics between the 2 groups (*P* > .05), but the untested group showed a significantly higher proportion of refractory peritonitis (*P* = .001) (Table 1).

**Table 1:** Clinical characteristics in the untested group and tested group based on thyroid function testing status.

Variables	Untested group (n* =* 101)	Tested group (n* =* 69)	*P* value
Sex (Male, n, %)	57 (56.44)	32 (46.38)	.20
Age (years)	56.65 ± 12.16	58.96 ± 11.60	.22
PD duration (months)	31.00 (15.00–48.00)	32.00 (17.50–47.50)	.82
Outcome (cure, n, %)	84 (83.17)	60 (86.96)	.50
Refractory peritonitis (n, %)	60 (59.41)	22 (31.88)	.001
ALB (g/L)	27.75 ± 4.53	28.51 ± 3.69	.25
Peritoneal effluent white cell count (10^9^/L)	3.30 (1.00–7.52)	2.40 (1.06–4.55)	.07
PCT (ng/ml)	9.12 (1.65–29.32)	2.85 (0.87–22.82)	.05
CRP (mg/L)	135.00 (78.35–182.00)	104.00 (37.40–174.00)	.09
WBC (10^9^/L)	11.00 ± 5.52	10.35 ± 4.44	.40

ALB = albumin, CRP = C-reactive protein, PCT = procalcitonin, PD = peritoneal dialysis, WBC = peripheral white blood cell.

**Figure 1.**
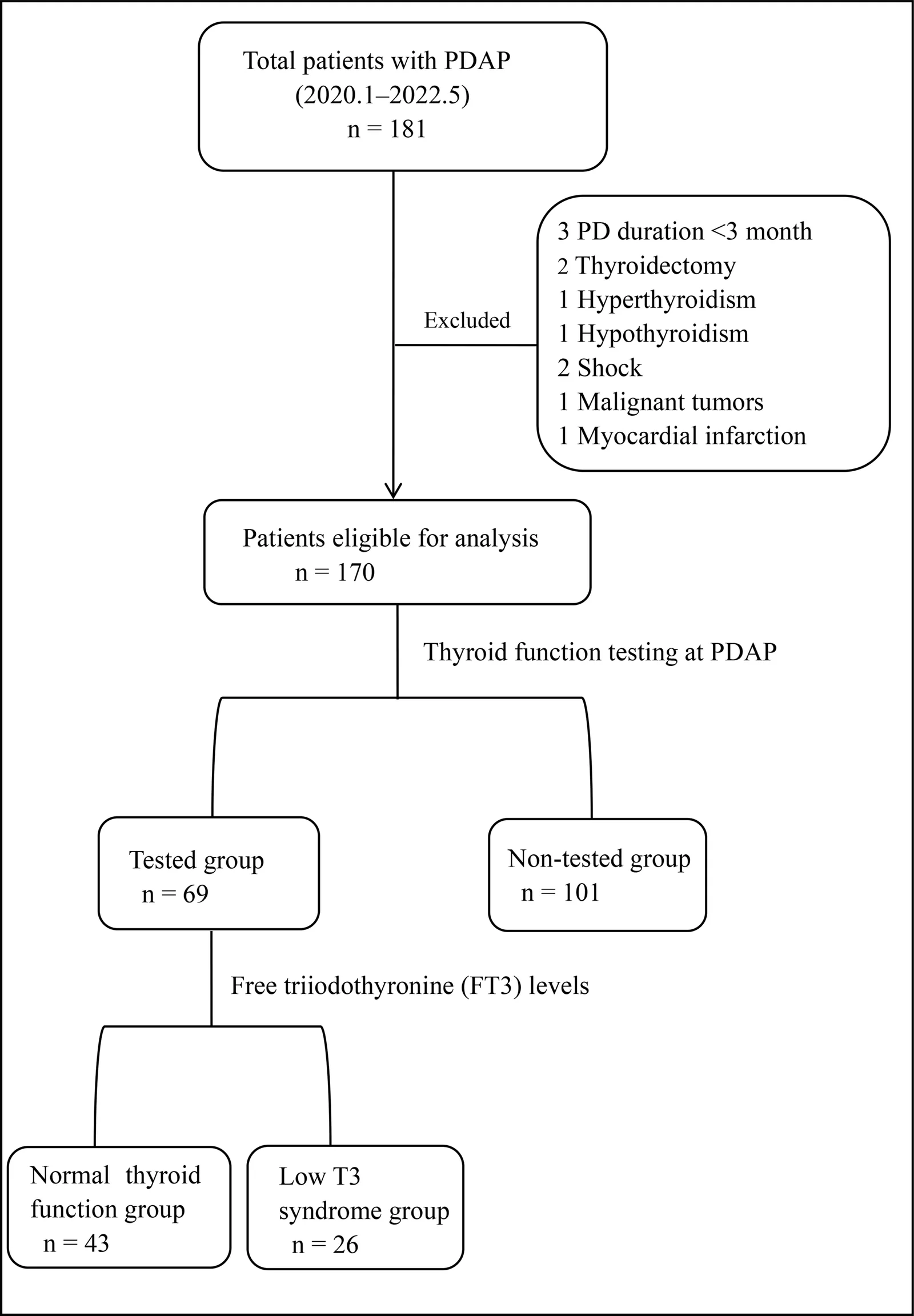
The flowchart shows the exclusion and selection of patients.

Among 69 patients who underwent thyroid function testing, 26 patients (37.68%) were classified into the low T3 syndrome group and 43 patients (62.32%) into the normal thyroid function group. The leading causes of ESRD included chronic nephritis (31.9%), diabetic nephropathy (30.4%), and hypertensive nephropathy (21.7%). Other causes included ANCA-associated nephritis, lupus nephritis, gouty nephropathy, obstructive nephropathy, and polycystic kidney disease. Peritoneal dialysis fluid culture results indicated that in the normal thyroid function group, there were 21 cases of Gram-positive bacteria, 7 cases of Gram-negative bacteria, 13 culture-negative cases, and 1 case each of tuberculosis and fungal infection. The low T3 syndrome group included 15 cases of Gram-positive bacteria, 5 cases of Gram-negative bacteria, 6 culture-negative cases, and no cases of tuberculosis or fungal infection.

### 3.1. Comparison of clinical indicators in two groups

Compared with the normal thyroid function group, the low T3 syndrome group showed a higher proportion of refractory peritonitis, higher peripheral WBC and neutrophil counts, as well as higher PCT and CRP levels, and lower baseline FT3 levels (all *P* < .05). There were no significant differences in other indicators (all *P* > .05) (Table 2).

**Table 2:** Clinical characteristics in the low triiodothyronine syndrome group and normal thyroid function group.

Variables	Normal thyroid function group (n* = *43)	Low T3 syndrome group (n* =* 26)	*P* value
Sex (Male, n, %)	22 (51.16)	10 (38.46)	.30
Age (years)	58.26 ± 11.74	60.12 ± 11.52	.52
PD duration (months)	31.00 (18.00–45.00)	34.5 (26.50–48.75)	.32
BMI (kg/m^2^)	24.02 ± 2.93	23.67 ± 2.63	.62
Outcome (cure, n, %)	36 (83.72)	24 (92.31)	.51
Refractory peritonitis (n, %)	7 (16.28)	10 (38.46)	.04
FT3 at PDAP (pmol/L)	3.73 ± 0.47	2.77 ± 0.39	<.001
FT4 at PDAP (pmol/L)	11.70 (9.28–13.29)	11.92 (10.57–12.40)	.69
TSH at PDAP (μIU/ml)	1.64 (1.05–2.98)	1.44 (0.93–2.38)	.31
Interval from baseline FT3 to PDAP onset (months)	8.00 (4.00–19.00)	7.50 (1.75–11.50)	.19
Baseline FT3 (pmol/L)	3.75 ± 0.62	3.41 ± 0.69	.04
Baseline FT4 (pmol/L)	10.71 (9.25–12.17)	10.45 (9.27–11.41)	.59
Baseline TSH (μIU/ml)	2.16 (1.19–2.97)	1.43 (0.99–2.13)	.10
Hemoglobin (g/L)	103.58 ± 24.94	100.81 ± 24.47	.65
ALB (g/L)	28.91 ± 3.57	27.85 ± 3.87	.25
Peritoneal effluent white cell count (10^9^/L)	2.24 (0.98–4.20)	2.52 (1.47–5.75)	.40
PCT (ng/ml)	1.73 (0.42–22.00)	6.67 (1.90–31.37)	.02
CRP (mg/L)	71.16 (25.00–146.00)	133.99 (101.23–187.75)	.03
WBC (10^9^/L)	9.19 ± 3.75	12.27 ± 4.87	.008
Neutrophil count (10^9^/L)	7.72 ± 3.78	10.81 ± 5.04	.01
NLR	9.61 (5.83–18.97)	14.43 (8.44–25.28)	.09
PLR	351.11 (203.65–502.30)	321.97 (215.6–558.95)	.89

ALB = albumin, BMI = body mass index, CRP = C-reactive protein, FT3 = free triiodothyronine, FT4 = free thyroxine, NLR = neutrophil-to-lymphocyte ratio, PCT = procalcitonin, PD = peritoneal dialysis, PDAP = peritoneal dialysis-associated peritonitis, PLR = platelet-to-lymphocyte ratio, T3 = triiodothyronine, TSH = thyroid stimulating hormone, WBC = peripheral white blood cell.

### 3.2. Association between clinical indicators and FT3 in peritonitis

Correlation analysis indicated that FT3 levels at peritonitis onset were negatively correlated with WBC (*r* = −0.370, *P* = .002), neutrophil count (*r* = −0.375, *P* = .001), PCT (*r* = −0.289, *P* = .016), CRP (*r* = −0.326, *P* = .006), NLR (*r* = −0.337, *P* = .005) and positively correlated with baseline FT3 levels (*r* = 0.417, *P* < .001) (Fig. 2A–F).

**Figure 2.**
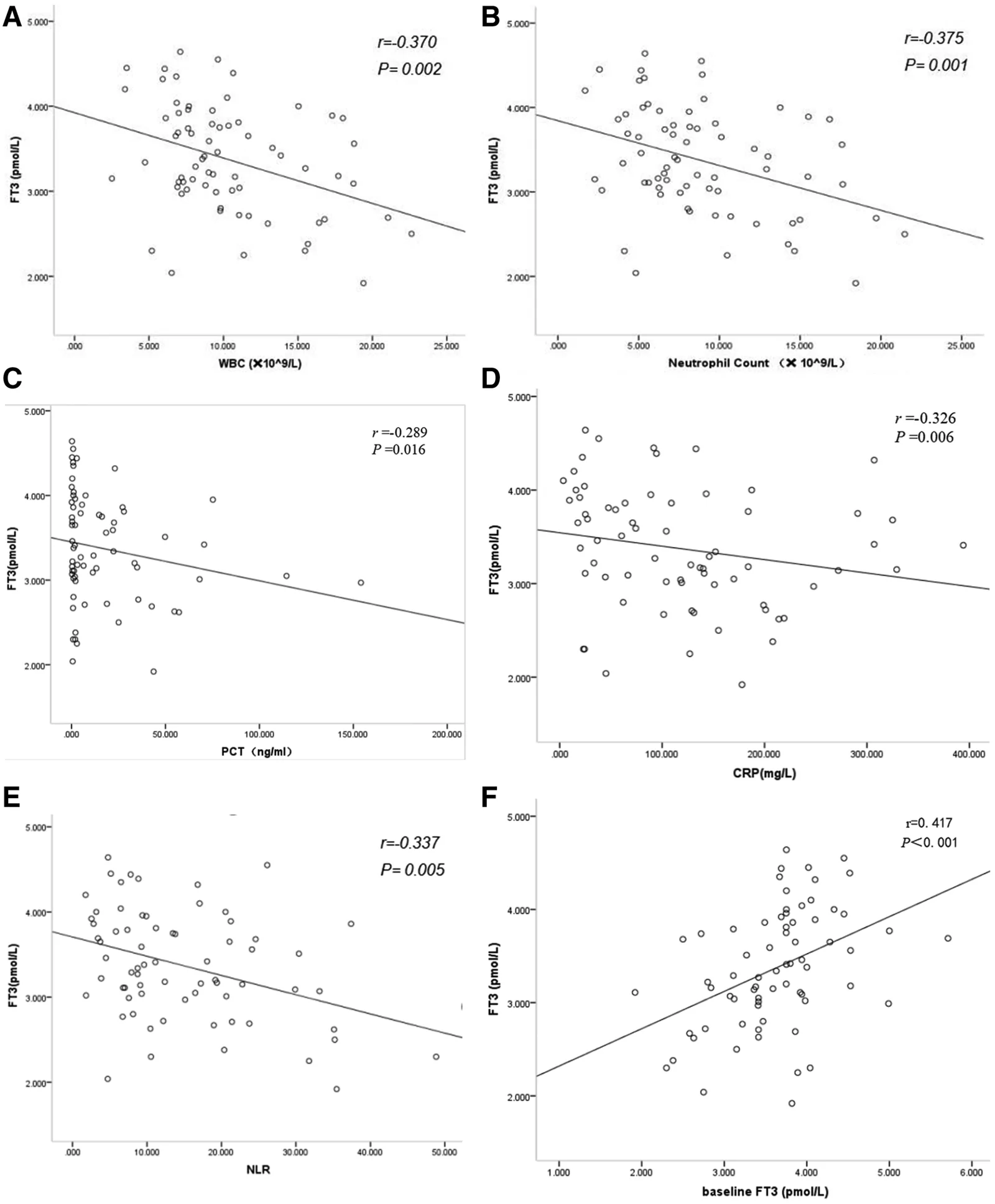
Univariate correlations between free triiodothyronine (FT3) at PDAP and peripheral WBC (white blood cell, A), neutrophil count (B), PCT (procalcitonin, C), CRP (C-reactive protein, D), NLR (neutrophil-to-lymphocyte ratio, E), and baseline FT3 (F).

Variables associated with FT3 at PDAP were analyzed using univariate and multivariate linear regression analyses. Univariate analysis results showed that baseline FT3 levels were positively correlated to FT3 levels at PDAP (*P* < .001), whereas WBC, neutrophil count, and NLR were negatively correlated with FT3 levels at PDAP (*P* = .002, *P* = .001 and *P* = .002, respectively) (Table 3).

**Table 3:** Univariate linear regression analysis of risk factors associated with free triiodothyronine at peritoneal dialysis-associated peritonitis.

Variables	Standardized β	Unstandardized B	95% CI	*P* value
Age (years)	−0.141	−0.008	−0.021 to –0.006	.25
PD duration (months)	−0.238	−0.006	−0.013 to –0.001	.05
BMI (kg/m^2^)	0.118	0.027	−0.028 to −0.082	.33
Baseline FT3 (pmol/L)	0.417	0.401	0.188 to –0.614	<.001
Hemoglobin (g/L)	−0.058	−0.002	−0.008 to –0.005	.64
ALB (g/L)	0.067	0.012	−0.030 to −0.054	.58
PCT (ng/ml)	−0.202	−0.005	−0.010 to –0.001	.09
CRP (mg/L)	−0.207	−0.001	−0.003 to –0.001	.09
WBC (10^9^/L)	−0.370	−0.053	−0.086 to −0.021	.002
Neutrophil count (10^9^/L)	−0.375	−0.053	−0.085 to −0.021	.001
NLR	−0.367	−0.023	−0.037 to −0.009	.002
PLR	−0.141	0.001	−0.001 to −0.001	.25

ALB = albumin, BMI = body mass index, CRP = C-reactive protein, FT3 = free triiodothyronine, NLR = neutrophil-to-lymphocyte ratio, PCT = procalcitonin, PD = peritoneal dialysis, PLR = platelet-to-lymphocyte ratio, WBC = peripheral white blood cell.

PD duration, PCT, CRP, baseline FT3, WBC, neutrophil count, and NLR, which showed *P* < .10 in univariate linear regression analysis, were included in the initial multivariate model. Using a stepwise method, only baseline FT3 and WBC in the final model remained as factors independently associated with FT3 levels at peritonitis onset (*B* = 0.410, 95% CI 0.214–0.605, β = 0.426, *P* < .001; *B* = −0.055, 95% CI −0.084 to −0.026, *β* = −0.380, *P* < .001) (Table 4). The analysis of variance results showed that the final multivariate linear regression model was significant (*F* = 15.404, *P* < .001). The residual-versus-predicted plot showed a random scatter around zero, supporting homoscedasticity, and the P-P plot showed points closely following the diagonal line, supporting approximate normality of residuals. Both VIF values were below 2.0, indicating no significant multicollinearity issues.

**Table 4:** Multivariate linear regression analysis of risk factors associated with free triiodothyronine at peritoneal dialysis-associated peritonitis.

Variables	Standardized β	Unstandardized B	95% CI	*P* value	VIF
Constants		2.455	1.681 to 3.229	<.001	
Baseline FT3 (pmol/L)	0.426	0.410	0.214 to 0.605	<.001	1.001
WBC (10^9^/L)	−0.380	−0.055	−0.084 to −0.026	<.001	1.001

FT3 = free triiodothyronine, VIF = variance inflation factor, WBC = peripheral white blood cell.

### 3.3. Preliminary discriminatory value of PDAP complicated by low T3 syndrome

ROC curve analysis was performed to evaluate the preliminary discriminatory ability, quantified by the area under the curve (AUC), of the combination of baseline FT3 and the inflammatory markers in identifying PDAP complicated by low T3 syndrome. The AUC values for combinations of baseline FT3 with WBC, neutrophil count, NLR, CRP, PCT, and PLR were 0.750 (95% CI 0.630–0.869, *P* =.001), 0.744 (95% CI 0.620–0.868, *P* = .001), 0.721 (95% CI 0.591–0.850, *P* =.002), 0.665 (95% CI 0.534–0.796, *P* =.022), 0.659 (95% CI 0.522–0.796, *P* = .028), and 0.640 (95% CI 0.500–0.779, *P* =.053), respectively (Fig. 3).

**Figure 3.**
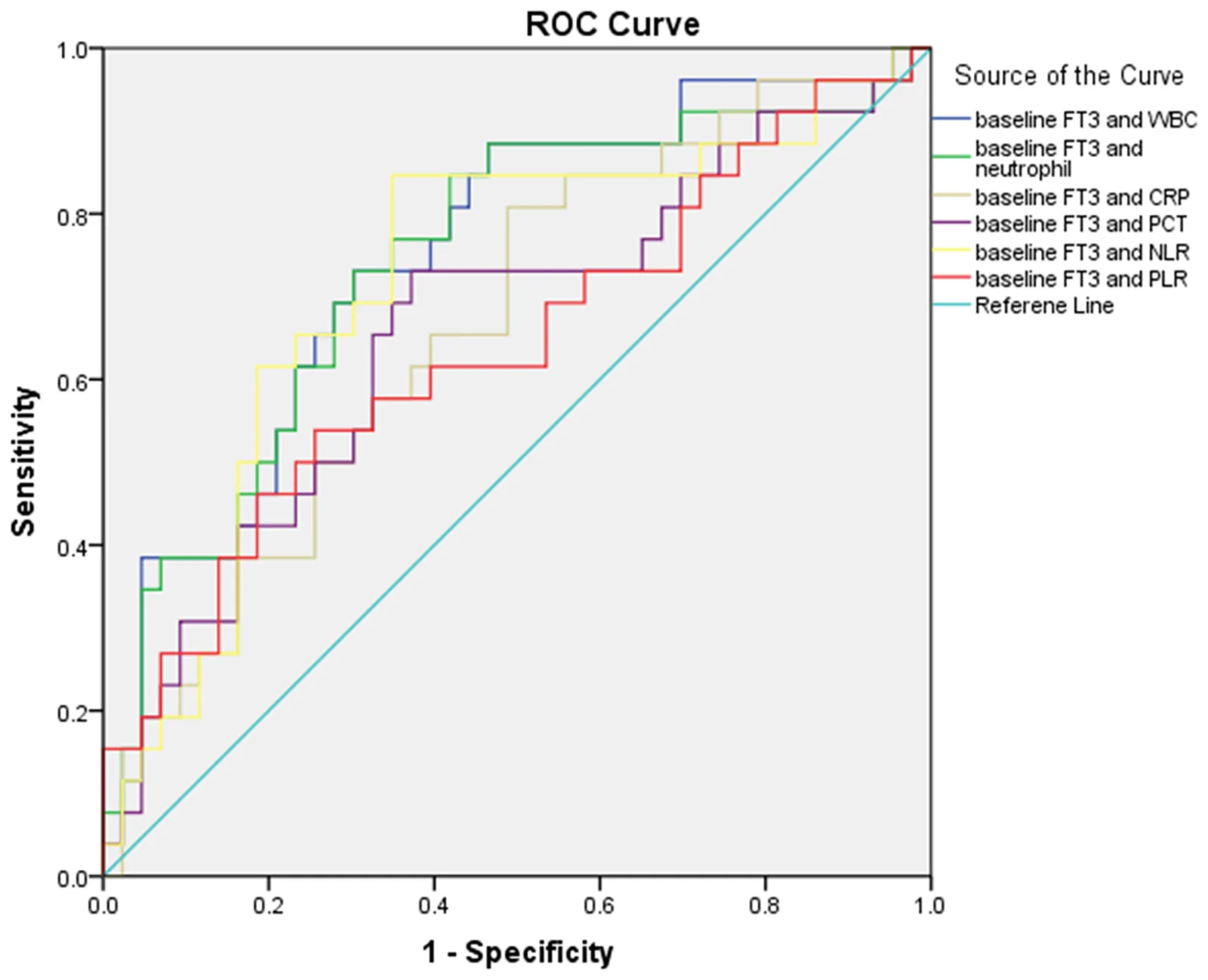
ROC curves based on the combination of baseline FT3 with WBC (white blood cell), baseline FT3 with neutrophil, baseline FT3 with PCT (procalcitonin), baseline FT3 with CRP (C-reactive protein), baseline FT3 with NLR (neutrophil-to-lymphocyte ratio), and baseline FT3 with PLR (platelet-to-lymphocyte ratio).

## 4. Discussion and conclusions

Low T3 syndrome is common in patients with chronic kidney disease and those undergoing dialysis. However, it is often overlooked by both clinicians and patients, and only a limited number of studies focus on this condition and its clinical implications. Oxidative stress, volume overload, loss of residual kidney function, and the biocompatibility of peritoneal dialysis fluid often contribute to microinflammation in dialysis patients.^[8]^ During peritonitis, the loss of albumin, thyroid hormone-binding proteins, and thyroid hormones in peritoneal effluent may result in low T3 levels and hypoalbuminemia. Zoccali C et al reported that low FT3 levels in hemodialysis patients were associated with elevated inflammatory markers (interleukin-6 and CRP) and endothelial activation markers. They also found that FT3 levels increased once infection was controlled, suggesting suppression by inflammatory mediators.^[9]^ Furthermore, the low T3 syndrome group exhibited significantly elevated levels of inflammatory markers during peritonitis, including peripheral WBC, neutrophil count, PCT, and CRP. These findings suggested that higher inflammation was associated with greater FT3 suppression, which was consistent with the aforementioned literature. Chang TI et al reported that PD patients with lower T3 levels were at a higher risk of anemia, hypoalbuminemia, and elevated CRP levels.^[10]^ In this study, although ALB and hemoglobin levels were lower in the low T3 syndrome group than in the normal thyroid function group, the differences were not statistically significant.

CRP, as an acute-phase response protein reflecting inflammation, is highly sensitive to inflammatory conditions but lacks specificity. CRP levels are also elevated in autoimmune diseases, acute myocardial infarction, and certain critical illnesses. PCT and interleukin-6 are better inflammatory markers for assessing the severity of early infections, but specific testing equipment may not be readily available in certain community hospitals. Therefore, other inflammatory markers that are more accessible and effective in clinical practice have attracted the attention of clinicians.

Okyay GU et al found that pre-dialysis, hemodialysis, or PD patients had higher NLR than healthy individuals.^[11]^ During inflammation, inflammatory factors triggered an increase in neutrophil counts and apoptosis of lymphocytes, leading to elevated NLR. High NLR predicted both all-cause and cardiovascular mortality in PD patients.^[12]^ A study enrolling 202 patients showed that higher NLR was associated with increased PDAP treatment failure, as evidenced by all-cause mortality and catheter removal rates.^[13]^ Another study indicated that NLR was an independent predictor of PDAP following catheter placement.^[14]^ Elevated PLR reflected the link between inflammation and increased platelet counts or decreased lymphocyte counts. Kultigin Turkmen et al demonstrated that both PLR and NLR could predict inflammatory responses in ESRD patients, with PLR showing better predictive efficacy than NLR.^[15]^ Dan Zhou et al found that NLR and PLR served as risk factors for PDAP treatment failure.^[16]^ A multicenter retrospective study showed that a higher risk of new PDAP was observed in the high PLR group than in the low PLR group.^[17]^ In this study, NLR and PLR were used as inflammatory markers to assess the inflammatory status in patients with PDAP complicated by low T3 syndrome. The results demonstrated that FT3 levels at peritonitis onset were negatively correlated with NLR, but not with PLR. Among inflammatory markers, WBC and baseline FT3 were independently associated with FT3 levels during peritonitis, and their combination showed the highest AUC values for identifying PDAP complicated by low T3 syndrome. Notably, combining baseline FT3 with WBC, neutrophil count, or NLR demonstrated numerically higher AUC values compared with models that incorporated baseline FT3 with either PCT or CRP.

Some studies have indicated that FT3 levels are associated with PD outcomes. Additionally, it could independently predict the mortality risk in PD patients.^[18]^ Our study found that PDAP patients complicated by low T3 syndrome had a higher proportion of refractory peritonitis. However, no significant difference in peritonitis outcomes was observed between the 2 groups. This could be explained by the small number of patients diagnosed with refractory peritonitis who did not choose to switch to hemodialysis. Instead, they received higher-level antibiotics or extended antibiotic treatment duration, ultimately meeting the cure criteria and increasing the cure rate. In clinical practice, if refractory peritonitis is not promptly managed by removing the peritoneal dialysis catheter or using prolonged multiple antibiotic therapies, the incidence of antibiotic-related adverse reactions, multidrug-resistant infections, and secondary or more serious complications may increase.

We acknowledged certain limitations of this retrospective single-center study. First, because of limited clinical attention to CKD complicated with low T3 syndrome, thyroid function has not been recommended as a regular screening indicator. Consequently, the exclusion of a significant number of patients with PDAP who lacked thyroid function data resulted in a relatively small sample size for this study. Second, the untested group had a significantly higher prevalence of refractory peritonitis, indicating potential selection bias, possibly attributable to physician-driven thyroid function testing. The testing was conducted at the time of peritonitis diagnosis, rather than being based on the outcome of peritonitis. However, physician-driven thyroid testing may have reduced comparability between the tested and untested groups. This should be considered an important limitation when interpreting the difference in refractory peritonitis rates. Third, the single-center design and small sample size may limit the generalizability of the study findings. The lack of internal validation restricted these findings to exploratory rather than confirmatory conclusions. The ROC analysis was only described as a preliminary assessment of discriminatory potential, rather than as presenting definitive predictive power. Finally, given that thyroid hormone levels fluctuate during PDAP, the values measured at hospital admission may not adequately reflect the overall trend over time. Therefore, we recommend that physicians perform thyroid function tests as part of routine screening. Prospective designs with larger sample sizes in multicenter studies are needed in future research to confirm these results.

In conclusion, low T3 syndrome is a latent complication in patients with PDAP, which is usually overlooked and may not be readily diagnosed by clinicians. Our study found that PDAP patients complicated by low T3 syndrome exhibited elevated levels of inflammatory markers and a higher proportion of refractory peritonitis. Furthermore, the combination of baseline FT3 and inflammatory markers demonstrated preliminary discriminatory value in identifying low T3 syndrome in patients with PDAP. As a novel, effective, and readily available inflammatory marker, the NLR, in combination with baseline FT3 measured at the time point closest to peritonitis onset, may serve as a valuable preliminary discriminatory marker for identifying PDAP complicated by low T3 syndrome. To the best of our knowledge, this was the first study to explore the relationship between low T3 levels and inflammation in this cohort. We hope that this study will encourage more physicians to heighten awareness of the relationship between low T3 syndrome and PDAP, so that relevant precautions and treatments can be implemented in future practical applications.

## Acknowledgments

We would like to thank Yuzhen Fang and Mingyi Xiao for their assistance in communicating with patients.

## Author contributions

**Supervision:** Jiaxin Liu.

**Writing – original draft:** Jiaxin Liu.

**Writing – review & editing:** Jiaxin Liu.

**Data curation:** Liqiang Lv.

**Validation:** Junyuan Xiao.

**Formal analysis:** Rui Wu.

**Methodology:** Rui Wu.

**Investigation:** Chuxin Chen.

**Project administration:** Yanqiang Peng.
